# The development and preliminary evaluation of a financial navigation program among patients with breast cancer in China

**DOI:** 10.1016/j.apjon.2025.100668

**Published:** 2025-02-17

**Authors:** Xiaoyi Yuan, Liqin Chen, Yanling Sun, Yi Kuang, Junyi Ruan, Lichen Tang, Jiajia Qiu, Weijie Xing

**Affiliations:** a School of Nursing, Fudan University, Shanghai, China; b Fudan University Shanghai Cancer Center, Shanghai, China; c School of Public Health, Fudan University, Shanghai, China

**Keywords:** Breast cancer, Financial navigation, Financial toxicity, Cost-related health literacy, Feasibility, Randomized controlled trial

## Abstract

**Objective:**

This study aims to develop a financial navigation program among patients with breast cancer in China and assess its feasibility, acceptability, and preliminary effects on cost-related health literacy and financial toxicity (FT).

**Methods:**

The Medical Research Council (MRC) framework were adopted to guide the development of the financial navigation program, providing a structured approach to complex intervention development and evaluation. It consisted of three phases: evidence identification via a scoping review, intervention content modeling through qualitative study analysis, and outcome mapping based on social stress theory. The feasibility, acceptability and primary effectiveness were examined in a single-center, assessor-blinded pilot randomized controlled trial with 26 recruited patients.

**Results:**

The financial navigation program consists of needs assessment, cost-related health education, resource/service referral and personalized counseling. The consent rate and a 1-month attrition rate for the feasibility study of the intervention were 55.9% and 7.7%, respectively. Most (91.7%) participants were satisfied with the program and perceived benefits. The intervention significantly improved cost-related health literacy, although no statistically significant between-group difference in FT was observed.

**Conclusions:**

The MRC framework serves as a useful scientific basis for developing financial navigation program with a culturally sensitive approach. The financial navigation program was feasible, acceptable, effective in improving cost-related health literacy and has the potential to enhance FT among patients with breast cancer in China.

**Trial registration:**

ClinicalTrials.gov Identifier NCT06355440.

## Introduction

Female breast cancer ranked as the second leading cause of cancer incidence worldwide in 2022,^1^ with approximately 357,200 new cases in China, accounting for 7.4% of all cancer cases.^2^ While advancements in cancer therapies have improved survival rates, these treatments impose significant financial burdens on patients.^3^ Financial toxicity (FT), defined as the economic burdens and distress associated with disease and treatment, has emerged as a critical issue, particularly for patients with breast cancer who often require long-term and multidisciplinary survivorship care.^4^ Studies have shown that patients with breast cancer in low- and middle-income countries face substantially higher FT (78.8%) compared to those in high-income countries (35.3%).^5^ This underscores the heightened risk faced by patients in countries like China, where rapid adoption of advanced therapies is increasing treatment costs. FT could lead to detrimental consequences, including material loss, anxiety, depression, cost-related nonadherence, and impaired quality of life.^3^^,^^6^ Addressing FT is not only crucial to reducing financial barriers but also to ensuring equitable, high-quality cancer care and preserving the well-being of patients with breast cancer.

Strategies across individual, interpersonal, organizational, community, and policy levels are needed to prevent and mitigate FT.^7^ At the patient and provider level, financial navigation (FN) is a promising intervention to help cancer patients cope with financial challenges across the cancer continuum.^8^^,^^9^ Previous scoping reviews suggested that an effective FN program included components such as identifying financial needs, improving cost-related health literacy, supporting shared-decision making, and referring to financial assistance resources.^10^ Cost-related health literacy was the ability to obtain, process, communicate, and use health-related cost information to make informed treatment decisions.^11^ Improving cost-related health literacy may be an effective and feasible intervention strategy for reducing FT, as it empowers patients to make informed financial decisions, seek financial assistance, and adhere to treatments without unnecessary financial stress.^11^

There were two completed studies preliminarily affirmed the feasibility of FN.^10^ However, both of these studies developed their intervention from the perspective of patients in US and employed a pre-post design without a control group, which limits their ability to assess the effectiveness of the interventions. Health care policies and economic environment discrepancies between US and other countries, such as China, which makes the situation unique and in need of tailored interventions.^12^ In China, although basic medical insurance covers approximately 95% of the population and critical illness insurance provides nearly 50% reimbursement for cancer treatment, patients still face high out-of-pocket expenses, especially for newer treatments not fully covered by medical insurance.^13^ In addition, urban-rural disparities in medical resources force patients to travel long distances for high-quality treatments, adding to the financial burden.^13^ In Chinese culture, there is a reluctance to discuss treatment cost, both in provider–patient interactions and within families. This leaves patients with unmet needs when it comes to communicating costs of cancer care.^14^ Therefore, there is an increasing need to develop and evaluate a FN program for breast cancer tailored to the Chinese health care context.

To date, there have been no studies focused specifically on FT interventions for cancer patients in China, nor any studies conducted in contexts with similar socio-economic environments. This gap highlights the urgent need for contextually relevant strategies to address FT within Chinese health care and economic systems. FN involving in several components requiring the active participation of patients and facilitators is considered a complex intervention. The Medical Research Council (MRC) framework is helpful in developing such a complex intervention to make it evidence-based and relevant to context.^15^ Therefore, guided by the updated MRC framework for complex interventions, this study aimed to develop a FN program tailored to the needs of Chinese cancer patients and to assess its feasibility, acceptability, and preliminary effects on cost-related health literacy and FT. The findings from this study will lay a foundation for future rigorous effectiveness evaluations and provide high-quality evidence for addressing FT in cancer care.

## Methods

### The development of intervention

Following the updated MRC framework,^15^ the FN program was developed in three stages: (1) evidence identification via a scoping review, (2) intervention content modeling through qualitative study analysis, and (3) outcome mapping based on social stress theory.

#### Evidence identification

A scoping review^10^ identified interventions for alleviating cancer-related FT was first conducted. Among 15 included studies, FN emerged as the most frequently used and promising approach to address FT. It typically consisted of four components, which were applied as the skeleton for our intervention: (1) assessment of FT and unmet financial needs; (2) health education to enhance cost-related health literacy; (3) individualized counseling; (4) referral to financial assistance resources and social supports. However, standardized implementation and effectiveness of such intervention remained uncertain.

#### Intervention content modeling

To tailor the intervention to participants' needs and identify available resources for reducing FT, secondary analysis of our previous qualitative data from 23 patients with breast cancer and 14 stakeholders was conducted.^13^ Based on these findings, the content of the FN program was constructed: (1) Needs Assessment Module: a brief introduction of FT and assessment of patients’ information needs using a self-designed cost-related health literacy questionnaire; (2) Cost-Related Health Education Module: topics regarding communication of cost, treatment-related expenses, health insurance policies, and family support; (3) Resource/Service Referral Module: timely referrals to financial assistance programs and clinical professionals when identify unaddressed problems; (4) Personalized Counseling Module: offering one-on-one counseling for cost-related issues and coping strategies.

#### Outcome mapping based on theory

The social stress theory was adopted as the theoretical framework to map anticipated outcomes and hypothetical relationships.^16^^,^^17^ This theory identifies stressors, mediators (e.g., social support and coping), and outcomes as key elements. Cancer diagnosis and treatment are the primary stressors causing FT, while the FN intervention serves as a mediator.^18^, ^19^, ^20^ By enhancing coping resources, social support, cost-related health literacy, shared decision-making ability and perceived stress, the intervention aims to reduce FT.^5^^,^^21^^,^^22^ Consequently, the intervention outcomes include cost-related health literacy, shared decision-making ability, perceived stress, and FT.

#### Intervention delivery

The intervention was delivered over four sessions using a hybrid mode, including face-to-face communication, phone calls, and peer support group: (1) Session 1 (Needs Assessment) was delivered face-to-face on the first day of admission and lasted 5–10 minutes. (2) Session 2 (Cost-Related Health Education) was delivered face-to-face on the second-third day after admission and lasted 15–20 minutes. A booklet designed in a Q&A format, and evaluated by an expert panel for accuracy, significance, and readability, was used as the educational material. (3) Session 3 (Resource/Service Referral) was delivered face-to-face or by phone from postoperative to 3 months after discharge when the navigator identifies unaddressed problems, lasting 10 minutes each time. (4) Session 4 (Personalized Counseling) was delivered via monthly follow-up phone calls and weekly WeChat-based peer support group within 3 months post-discharge, lasting 10 minutes each time. The delivery of FN intervention is presented in Table 1.

**Table 1:** Detailed content of the financial navigation intervention for breast cancer patients.

Intervention modules	Delivery time	Contents	Duration	Delivery format	Location
**Session 1** Needs assessment module	1^st^ day of admission	1 Introduce cancer-related FT, its causes and risk factors 2 Highlight the potential negative effects of FT 3 Assess information needs using the cost-related health literacy questionnaire	5–10 min	Face-to-face communication booklet	Breast surgery ward
**Session 2** Cost-related health education module	2^nd^-3^rd^ day after admission	1 Teach how to communicate with doctors about costs of cancer treatment 2 Teach how to keep track of treatment-related expenses 3 Provide information on health insurance policies 4 Enhancing awareness against medical fraud 5 Provide advice on realigning household responsibilities and making a family financial budget	15–20 min	Face-to-face, tailored education booklet	Breast surgery ward
**Session 3** Resource/Service referral module	From postoperative to 3 months after discharge	1 Navigate to access financial assistance information and resources 2 Refer to psychologists when identify severe negative emotions 3 Refer to physicians when identify severe adverse effects of treatment	10 min	Face-to-face communication /Telephone follow-up booklet	Breast surgery ward Off-hospital
**Session 4** Personalized counseling module	Monthly after discharge	1 Provide information on symptom management and how to return normal life and work 2 Assess maladaptive behaviors due to cost concern 3 Encourage coping skill exchange for FT within WeChat-based peer support group 4 Provide counseling for personalized cost-related issues	10 min	Telephone follow-up WeChat	Off-hospital

FT, financial toxicity.

### The preliminary evaluation of intervention

A single-center, assessor-blinded pilot randomized controlled trial was conducted to examine the feasibility, acceptability, and preliminary effects of FN program among patients with breast cancer. The study was prospectively registered on ClinicalTrials.gov (NCT06355440).

#### Setting and participants

The study was conducted at a cancer center in Shanghai, China. Participants were recruited from a breast surgery ward in April 2024. Inclusion criteria included (1) female, 18 years or older; (2) newly diagnosed with breast cancer and undergoing surgery during this hospitalization; (3) considering that patients receiving adjuvant therapy experience more significant FT, only those who are receiving or expecting to undergo at least one adjuvant treatment (e.g., chemotherapy, radiotherapy, targeted therapy) following surgery are included; (4) Eastern Cooperative Oncology Group Performance Status (ECOG-PS) of 0–2; and (5) provided informed consent. Exclusion criteria included (1) diagnoses of ductal carcinoma in situ (DCIS), which treatment’ costs are almost entirely covered by basic medical insurance, resulting in minimal FT for patients; (2) serious psychiatric disorders; (3) cognitive impairments; or (4) difficulty in reading, writing, or communicating in Chinese.

#### Sample size

As a rule of thumb, a minimum of 10 participants per group is required to evaluate feasibility and estimate preliminary effects.^23^ Accounting for potential attrition, a total of 26 patients (13 per group) were enrolled in this pilot study.

#### Recruitment, randomization, and allocation

Convenience sampling was used to recruit participants for the study. The principal investigator (PI) identified potential participants from the hospital's electronic medical records, ensuring that they met the study's inclusion criteria. Eligible participants were then approached by the PI, who provided detailed information about the study's purpose, procedures, and potential risks. Participants were given the opportunity to ask questions and were allowed sufficient time to consider their participation. Written informed consent was obtained from all participants who agreed to participate.

To avoid contamination between patients, a cluster randomization design was employed, with four-person rooms in the breast surgery ward as the randomization units. The rooms in the ward were sequentially numbered from 1 to 12 based on bed number in ascending order. A research assistant, not involved in recruitment or data collection, generated the random sequence using an online random number generator (https://www.random.org/). As a result, rooms 1, 4, 5, 6, 7, and 10 were assigned to the intervention group, while rooms 2, 3, 8, 9, 11, and 12 were assigned to the control group. According to room numbers, participants were randomly allocated to the intervention or control group in a 1:1 ratio. Given the nature of the intervention, it was not feasible to blind participants and intervention personnel. Blinding was only implemented for the outcome assessors in this study.

#### Intervention

Participants in the intervention group received both the FN intervention and usual care, whereas control group participants received only usual care. To ensure the fidelity of the intervention, all sessions were conducted by a well-prepared nurse with extensive experience in breast cancer care as well as substantial knowledge of FT. She also played a central role in the development of the intervention, ensuring consistency in its delivery and adherence to the designed protocol. During the face-to-face intervention phase, a supervisor conducted periodic observations to ensure the intervention was delivered consistently and followed the protocol. During the online communication phase, the supervisor also joined the WeChat-based peer support group that included both the intervener and the participants, allowing for real-time monitoring of the intervention content and quality, ensuring fidelity throughout the process.

Participants in the control group received usual care, which included routine inpatient and discharge education covering topics such as an introduction to the ward environment, preoperative preparation, wound care, upper limb functional exercises, nutrition, emotional support, follow-up appointments, and complications signs. Financial-related questions were answered by their doctors and nurses, but no comprehensive education or follow-up was provided on this topic.

#### Outcomes

Given the short follow-up period (1 month) of this pilot study, changes in behaviors or long-term health outcomes might be difficult to observe.^24^ Thus, this pilot study evaluated feasibility, acceptability, and preliminary effects on cost-related health literacy and FT of the intervention.

Feasibility was assessed by participants’ recruitment, attrition, and compliance, including eligibility, consent, attrition, and adherence rate. Eligibility rate was the proportion of participants meeting eligibility criteria among those assessed for eligibility. Consent rate was the proportion of eligible participants who agreed to participate among those who were eligible for participation. Attrition rate was the percentage of participants who dropped out after enrolment. Adherence rate was the proportion of participants completing the entire program. Reasons for ineligibility, refusal, dropout, and nonadherence were recorded.

Acceptability was assessed via feedback from intervention group participants through a self-designed satisfaction questionnaire and semi-structured interviews. The five-item questionnaire collected participants' satisfaction with the content, mode, frequency, duration, and perceived usefulness of the intervention, rated on a five-point scale from 1 (strongly disagree) to 5 (strongly agree). Higher scores indicated higher satisfaction. The questionnaire was reviewed and revised by five experts, showing the satisfactory content validity index (CVI) of 0.92. One-on-one interviews explored participants’ experiences with the intervention and suggestions for improvement by phone.

Cost-related health literacy was measured by a self-developed questionnaire, based on a concept analysis, as no existed tool was available.^25^ The questionnaire consists of 10 statements, including 2 items on cost discussing cost, 4 items on understanding health-related information, and 4 items on obtaining or applying financial assistance. Participants rated statements on a four-point scale from 1 (strongly disagree) to 4 (strongly agree), with higher scores indicating higher cost-related health literacy. The questionnaire was reviewed and revised by five experts from diverse fields, including oncology treatment, nursing, health insurance policy, social work, and hospital management, demonstrating a CVI of 0.90.

FT was evaluated using multidimensional measurement indicators, addressing material, psychological, and behavioral aspects, as highlighted by several systematic reviews.^18^^,^^19^ Material hardship was assessed with two questions adapted from the Medical Expenditure Panel Survey (MEPS)^26^: (a) substantial depletion of savings, or selling assets, and (b) incurrence of debt due to treatment-related expenses in the past month. Psychological response was measured by the COmprehensive Score for Financial Toxicity-Functional Assessment of Chronic Illness Therapy Version 2 (COST-FACIT; Version 2), a 12-item validated self-report measure (Cronbach's *α* ​= ​0.86) developed by de Souza et al., in 2014 and translated into Chinese by Chan et al., in 2021.^27^^,^^28^ Participants rated items on a 5-point scale over the past 7 days, ranging from 0 (not at all) to 4 (very much), with lower scores (range 0–44) indicating greater FT. Behavioral change was assessed by employment changes and cost-related nonadherence with six questions adapted from MEPS^26^ and previous literature^29^, ^30^, ^31^ on delaying, foregoing or changing treatments due to cost concerns in the past month.

#### Data collection

Data was collected from April 1st 2024 to May 8th 2024. At baseline, participants' sociodemographic and medical information, COST-FACIT (Version 2), and cost-related health literacy were collected via paper questionnaires after obtaining written informed consent. At 1-month post-intervention, a research assistant who did not participate in the intervention collected participants' treatment information, material hardship, COST-FACIT (Version 2), cost-related behavioral changes, and cost-related health literacy by phone. During the week following the 1-month follow-up, all participants in the intervention group were individually interviewed to provide feedback on their experiences with the intervention. The interviews were conducted by the research assistant to gather qualitative data regarding the experience of attending the program. The participants’ responses were recorded and transcribed for analysis.

### Data analysis

Percentages described eligibility, consent, attrition, and adherence rates. Satisfaction scores were summarized using median and interquartile range (P25, P75).

Quantitative data were entered using Epidata 3.1, analyzed with IBM SPSS 26.0, using the last observation carried forward (LOCF) method for missing data under the intention-to-treat (ITT) principle.^32^ Descriptive analyses included mean (standard deviation), frequency (percentage) for sociodemographic, clinical information, behavioral changes, COST-FACIT, and cost-related health literacy. Baseline equivalence between groups was tested using independent samples *t*-tests, chi-square tests, or *Fisher* exact test. Paired samples *t*-tests and independent samples *t*-tests assessed within-group changes and between-group differences in COST-FACIT and cost-related health literacy questionnaire. Hedges' g effect sizes (with 95% confidence intervals) were calculated based on change scores of COST-FACIT and cost-related health literacy between two groups. *Fisher* exact test compared binary variables (e.g., employment changes and cost-related nonadherence) between groups, with odds ratios (95% CI) estimating post-intervention effect size. All statistical tests were two-sided, with a significance level of *P* ​< ​0.05.

Qualitative data from interviews were transcribed verbatim and analyzed using content analysis guided by an inductive approach.^33^ First, two researchers independently read the transcripts multiple times to gain a comprehensive understanding of the data. Then, open coding was performed, and initial codes were assigned to meaningful segments of text. These codes were reviewed, compared, and grouped into categories and subcategories based on thematic similarities. Discrepancies in coding were discussed and resolved through consensus among the research team. NVivo 12 software was used to facilitate data organization and ensure systematic analysis.

### Ethical considerations

The study was conducted in accordance with the Declaration of Helsinki and received approval from the Ethics Committee of Fudan University Shanghai Cancer Center (Approval No. 2312288-16). The study protocol, including the participant recruitment and informed consent procedures, was reviewed and approved by the Ethics Committee. Written informed consent was obtained from all participants after they were fully informed of the study's objectives, procedures, potential risks, and their right to withdraw at any time without penalty. To ensure participant confidentiality, all data were securely stored in an anonymized format and access was restricted to authorized personnel only.

## Results

### Characteristics of the participants

The mean age of participants was 47.35 ​± ​10.23 years. Most were married (24 [92.3%]), and only eight (33.3%) had financially independent adult children. The majority (22 [84.6%]) had at least a senior high school education. All participants’ caregivers were their relatives. Family savings varied, with most participants (21 [80.8%]) having less than 200,000 RMB. All participants had basic medical insurance, while only seven (26.9%) had private insurance. For most participants (17 [70.8%]), the major source of medical expenses was basic medical insurance.

Most participants were diagnosed with invasive breast cancer (24 [92.3%]), at stage II/III (19 [73.1%]), and only three (11.5%) had other non-communicable diseases. Surgical procedures included breast-conserving surgery (13 [50.0%]), modified radical mastectomy (3 [11.5%]), tumor resection (4 [15.4%]), and reconstructive surgery (6 [23.1%]). At 1-month follow-up, most participants were receiving chemotherapy (15 [51.7%]), or targeted therapy (7 [24.1%]). Aside from age differences (*P* ​= ​0.021), no statistically significant differences in sociodemographic and clinical characteristics were found between groups. The overview of participants’ characteristics is presented in Table 2 and Table 3.

**Table 2:** The sociodemographic characteristics of participants (*N* ​= ​26).

Variables	All sample (*n* ​= ​26)	Intervention group (*n* ​= ​13)	Control group (*n* ​= ​13)	Statistical values	*P*-value
**Age, yr, Mean ​± ​SD**	47.35 ​± ​10.23	42.85 ​± ​7.99	51.85 ​± ​10.50	*t* ​= ​−2.460	0.021
**Residence**					
Shanghai	8 (30.8%)	4 (30.8%)	4 (30.8%)	–a	1.000
Other provinces	18 (69.2%)	9 (69.2%)	9 (69.2%)		
**Education level**					
Junior high school or below	4 (15.4%)	1 (7.7%)	3 (23.1%)	1.281^a^	0.667
Senior high school or Junior college education	8 (30.8%)	4 (30.8%)	4 (30.8%)		
University / college or high	14 (53.8%)	8 (61.5%)	6 (46.2%)		
**Marital status**					
Unmarried	2 (7.7%)	1 (7.7%)	1 (7.7%)	–a	1.000
Married	24 (92.3%)	12 (92.3%)	12 (92.3%)		
**Having children**					
No	2 (7.7%)	1 (7.7%)	1 (7.7%)	–a	1.000
Yes	24 (92.3%)	12 (92.3%)	12 (92.3%)		
**Children's status (*n*=24)**					
All are dependents	12 (50.0%)	7 (58.3%)	5 (41.7%)	3.159^a^	0.261
Some are adults but financially dependent	4 (16.7%)	3 (25.0%)	1 (8.3%)		
All are adults and financially independent	8 (33.3%)	2 (16.7%)	6 (50.0%)		
**Caregivers**					
Relatives	26 (100.0%)	13 (100.0%)	13 (100.0%)	–a	1.000
Others	0 (0.0%)	0 (0.0%)	0 (0.0%)		
**Having religion**					
No	26 (100.0%)	13 (100.0%)	13 (100.0%)	–a	1.000
Yes	0 (0.0%)	0 (0.0%)	0 (0.0%)		
**Family savings (in 10,000 RMB)**					
0–10	12 (46.2%)	8 (61.5%)	4 (30.8%)	*χ**^2^* ​= ​3.465	0.322
11–20	9 (34.6%)	4 (30.8%)	5 (38.5%)		
21–50	3 (11.5%)	1 (7.7%)	2 (15.4%)		
> 50	2 (7.7%)	0 (0.0%)	2 (15.4%)		
**Having basic medical insurance**					
Yes	26 (100.0%)	13 (100.0%)	13 (100.0%)	–a	1.000
No	0 (0.0%)	0 (0.0%)	0 (0.0%)		
**Having private insurance**					
No	19 (73.1%)	9 (69.2%)	10 (76.9%)	–a	1.000
Yes	7 (26.9%)	4 (30.8%)	3 (23.1%)		
**Major sources of medical expenses at 1-month follow-up (*n*=24)**^b^					
Out-of-pocket	6 (25.0%)	2 (16.7%)	4 (33.3%)	1.657^a^	0.640
Basic medical insurance	17 (70.8%)	9 (75.0%)	8 (66.7%)		
Public funding	1 (4.2%)	1 (8.3%)	0 (0.0%)		

a *Fisher* exact test.

b Two participants dropped out at the 1-month follow up.

**Table 3:** The clinical characteristics of participants (*N* ​= ​26).

Variables	All sample (*n* ​= ​26)	Intervention group (*n* ​= ​13)	Control group (*n* ​= ​13)	*χ**^2^* value	*P*-value
**Type of breast cancer**					
Invasive breast cancer	24 (92.3%)	13 (100.0%)	11 (84.6%)	–a	0.240
Others	2 (7.7%)	0 (0.0%)	2 (15.4%)		
**Breast cancer surgery**					
Breast-conserving surgery	13 (50.0%)	6 (46.1%)	7 (53.8%)	1.520^a^	0.792
Modified radical mastectomy	3 (11.5%)	1 (7.7%)	2 (15.4%)		
Tumor resection	4 (15.4%)	3 (23.1%)	1 (7.7%)		
Reconstructive surgery	6 (23.1%)	3 (23.1%)	3 (23.1%)		
**Stage of cancer**					
I	3 (11.5%)	1 (7.7%)	2 (15.4%)	1.445	0.927
II	10 (38.5%)	6 (46.1%)	4 (30.8%)		
III	9 (34.6%)	4 (30.8%)	5 (38.4%)		
IV	2 (7.7%)	1 (7.7%)	1 (7.7%)		
Unclear	2 (7.7%)	1 (7.7%)	1 (7.7%)		
**Having other non-communicable diseases**					
No	23 (88.5%)	12 (92.3%)	11 (84.6%)	–a	1.000
Yes	3 (11.5%)	1 (7.7%)	2 (15.4%)		
**Current treatment at 1-month follow-up (*n*=24)**^b^					
Chemotherapy	15 (62.5%)	9 (75.0%)	6 (50.0%)	–a	0.400
Targeted therapy	7 (29.2%)	4 (33.3%)	3 (25.0%)	–a	1.000
Hormone therapy	2 (8.3%)	0 (0.0%)	2 (16.7%)	–a	0.478
None	5 (20.8%)	2 (16.7%)	3 (25.0%)	–a	1.000

a *Fisher* exact test.

b Two participants dropped out at the 1-month follow up. This is a multiple-choice question - select one or more answer choices.

### Feasibility

#### Recruitment

**Fig. 1:**
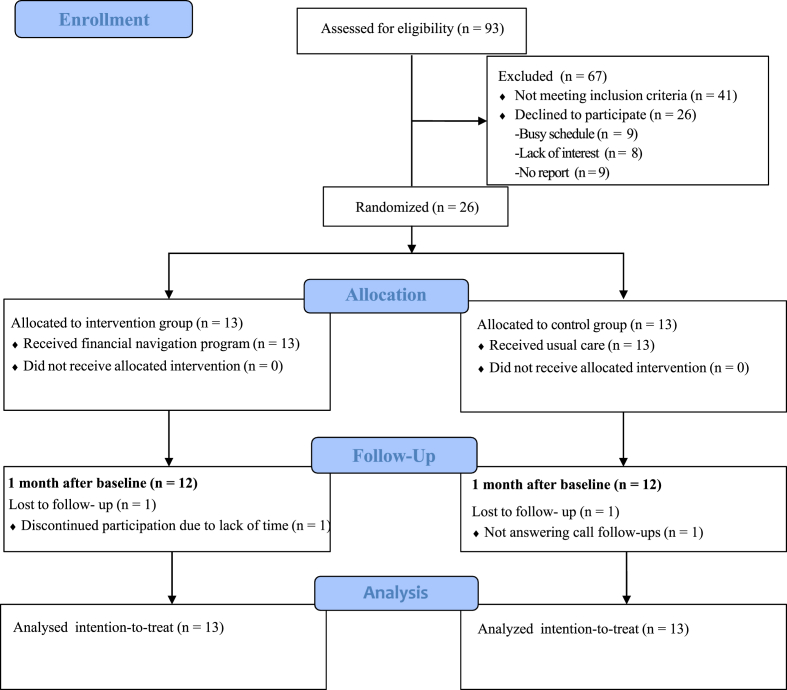
Flow diagram of participants' recruitment, allocation, and attrition.

A total of 93 patients with breast cancer were assessed for eligibility. Among them, 41 did not meet eligibility criteria (*n* ​= ​41; 21 had a non-cancer diagnosis, 13 had DCIS, 3 had recurrent breast cancer, and 4 did not undergo surgery during this hospitalization), resulting in an eligibility rate of 55.9%. Of the 52 eligible patients, 26 refused to participate due to busy schedules (9 [34.6%]), lack of interest (8 [30.8%]), or unspecified reasons (9 [34.6%]). Thus, 26 patients with breast cancer agreed to participate and signed informed consent, giving a consent rate of 50.0%. A flow diagram of participants’ recruitment, allocation, and attrition is shown in Fig. 1.

#### Attrition

One participant from the intervention group dropped out due to time constraints, and one from the control group was lost to follow-up due to loss of contact, resulting in an attrition rate of 7.7% (2/26).

#### Adherence

All participants completed baseline data collection, and 24 completed the post-intervention assessment (first month since baseline). All intervention group participants completed needs assessment and cost-related health education modules. Four participants (30.8%) reported that they did not review the booklet after the navigator introduced educational materials with them during hospitalization. The navigator proactively informed all intervention group participants about available resources or services, with three participants (23.1%) seeking more details on financial assistance, and two (15.4%) were referred to a psychologist for moderate to severe anxiety. For personalized counseling, four participants (30.8%) asked about rehabilitation, and six (46.2%) consulted personalized cost-related issues.

### Acceptability

The median satisfaction score in the intervention group (*n* ​= ​12) was 23.5 (21.5, 24.8). As shown in Table 4, almost all participants found the booklet helpful (91.7%), follow-up and personalized counseling useful (83.3%), and the frequency and duration appropriate (91.7%). Most (91.7%) participants were satisfied with the program and perceived benefits.

**Table 4:** Results of participants’ perceptions of the intervention (*N* ​= ​12)^a^.

	Strongly agree/Agree
1. The booklet is helpful for me to alleviate FT.	11 (91.7%)
2. The monthly follow-up phone call and personalized counseling are useful.	10 (83.3%)
3. The frequency and duration of this program are appropriate.	11 (91.7%)
4. Overall, participation in this program has been beneficial to me.	11 (91.7%)
5. Overall, I am satisfied with the program.	11 (91.7%)

a One participant dropped out at the 1-month follow up.

Twelve participants in the intervention group completed interviews, revealing two main categories: perceived benefits and suggestions (Table 5). Participants found the program provided useful knowledge to meet information needs, navigated them to access information and resources, promoted positive coping strategies, and made them feel supported. Suggestions for improvement included enriching information and delivery methods.

**Table 5:** Results of the qualitative interviews (*N* ​= ​12)^a^.

Categories	Subcategories	Quotations
Perceived benefits	Provide useful knowledge to meet information needs	"I am confused by the abundance of information online. This program offers professional and easily understandable information, helping to alleviate my financial burden." (P3) "The program provides valuable health-related cost information, helping me prepare for potential financial changes." (P10) "I knew little about my health insurance before. After joining this program, I understand how to use critical illness insurance to save money." (P8)
	Navigate to access information and resources	"The nurse navigator answered my personalized questions via WeChat after discharge, which helped me a lot." (P7) "This program taught me where to seek financial assistance resources, such as from the residents' committee and women's federation." (P9) "After reading the booklet, I knew where and how to apply for medical subsidies and successfully obtained them." (P2)
	Promote positive coping strategies	"Learning how to talk to my doctor about treatment costs helped me make informed decisions, which reduced my financial anxiety." (P5) "The advice on managing budget and realigning household responsibilities helped me adjust to changes, which made everything feel more manageable." (P11) "Having personalized counseling to discuss my symptoms and financial issues made me feel supported and more confident in dealing with challenges." (P4)
	Feel cared for and supported	"This program is patient-centered, and making me feel valued." (P6) "The nurse navigator is responsible and patiently answers my questions. Her professional support makes me feel comfortable." (P8)
Suggestions	Further enrich the information	"I am worried about recurrence and hope to receive professional advice on diet and exercise during rehabilitation." (P6) "It would be better to provide more professional knowledge about medical insurance, financial assistance programs, and disease management via WeChat." (P8) "Knowledge on self-care skills during rehabilitation should also be included in the booklet." (P1)
	Diverse delivery of information	"Presenting specific and vivid cases might enhance understanding of the information." (P11) "I would prefer to receive this information through short videos." (P7, P12)

a One participant dropped out at the 1-month follow up.

### Preliminary effects

#### Financial toxicity and cost-related health literacy

At baseline, the intervention group had lower FT scores (*P* ​= ​0.049, a lower score indicates a greater FT) than the control group. After the intervention, FT worsened in both intervention and control groups (*P* ​= ​0.039 and 0.024, respectively), with no statistically significant between-group difference (effect size ​= ​0.178, 95%CI: −0.60-0.94*, P* ​= ​0.483). At baseline, cost-related health literacy showed no statistically significant difference between groups (*P* ​= ​0.212). After the intervention, the intervention group's cost-related health literacy increased (*P* ​< ​0.001) and was higher than that of control group (effect size ​= ​1.216, 95%CI: 0.38–2.05*, P* ​= ​0.139). The pre- and post-intervention FT and cost-related health literacy are presented in Table 6.

**Table 6:** Financial toxicity and cost-related health literacy of participants (*N* ​= ​26)^a^.

Variables	Intervention group (*n* ​= ​13)	Control group (*n* ​= ​13)	*t* value^b^	*P*-value	Effect size^d^ (95% CI)
**Financial toxicity**					
Baseline	22.38 ​± ​5.46	26.77 ​± ​5.33	−2.074	0.049	0.178 (−0.60-0.94)
Post-intervention	17.54 ​± ​10.50	20.46 ​± ​10.42	−0.712	0.483	
*P*-value^c^	0.039	0.024			
**Cost-related health literacy**					
Baseline	20.46 ​± ​4.72	23.62 ​± ​7.46	−1.289	0.212	1.216 (0.38–2.05)
Post-intervention	29.46 ​± ​5.75	25.69 ​± ​6.76	1.531	0.139	
*P*-value^c^	< 0.001	0.241			

a Missing data were addressed using the last observation carried forward (LOCF) method following the intention-to-treat (ITT) principle.

b Between-group comparison by using two independent samples *t* test.

c Within-group comparison by using paired samples *t*-tests.

d Hedges'g effect size estimated based on the change score between two groups.

#### Behavioral change of FT

No significant between-group differences were found for debt or significant asset changes due to costs (*RR* ​= ​1.000, *P* ​= ​1.000). The intervention group had lower cost-related nonadherence (0.0%) compared to the control group (16.7%), though the difference was not statistically significant (*RR* ​= ​0.455, *P* ​= ​0.478). No significant between-group differences were found for employment changes (*RR* ​= ​0.467, *P* ​= ​0.214). Participants’ behavioral change of FT at 1-month follow up is presented in Table 7.

**Table 7:** Behavioral change of FT (*N* ​= ​24)^a^.

Variables	All sample (*n* ​= ​24)	Intervention group (*n* ​= ​12)	Control group (*n* ​= ​12)	*OR* (95% CI)	*P-value*
**Having debt/significant asset change due to costs**					
No	18 (75.0%)	9 (75.0%)	9 (75.0%)	1.000 (0.158–6.346)	1.000
Yes	6 (25.0%)	3 (25.0%)	3 (25.0%)		
**Have delayed/foregone/changed treatments due to costs**					
No	22 (91.7%)	12 (100.0%)	10 (83.3%)	1.200 (0.932–1.546)	0.478
Yes	2 (8.3%)	0 (0.0%)	2 (16.7%)		
**Employment changes**					
No changes	10 (41.7%)	3 (25.0%)	7 (58.3%)	0.238 (0.042–1.355)	0.214
Reduction in working time/unemployment	14 (58.3%)	9 (75.0%)	5 (41.7%)		

*OR*, Odds ratio.

a Two participants dropped out at the 1-month follow up.

## Discussion

This study developed a FN program and evaluated its feasibility, acceptability, and preliminary effects of for improving cost-related health literacy and alleviating FT among patients with breast cancer in China. Based on a scoping review, qualitative study, and social stress theory, the program was developed following the MRC framework for complex interventions. Our findings indicate that this program was feasible and acceptable, significantly enhancing cost-related health literacy, though no statistically significant between-group difference in FT was observed.

Guided by MRC framework,^15^ the development of this intervention comprehensively considered both best evidence and the local context. The framework of the FN was informed by systematic reviews,^10^ while the specific content was derived from interviews with patients and stakeholders in the local context.^13^^,^^14^ China's health care system and socio-economic environment differ significantly from those in Western countries, leading to unique FT challenges faced by cancer patients.^13^^,^^14^ For instance, Chinese patients often prioritize treatment effectiveness over cost, even to the extent of depleting all family savings. Additionally, cultural barriers make discussions about financial matters with health care providers or even within families difficult, resulting in unmet decision support needs. Regional disparities in resources lead to frequent cross-regional health care utilization, creating knowledge gaps in the use of out-of-area medical insurance. By tackling these specific challenges, the intervention offers a tailored approach to mitigating FT in the unique context of Chinese cancer care.

Feasibility of the intervention was supported by recruitment, attrition, and adherence. The consent rate of this pilot study was 50.0%, with common reasons for declining participation being busy schedules (34.6%) and lack of interest (30.8%). This might be attributed to the overwhelming nature of a breast cancer diagnosis, leaving patients anxious and unable to focus on the program early in their care.^34^ Expert consensus suggested intervening proactively at the diagnosis or before treatment held great potential to mitigate FT.^35^ To increase the consent rate for future implementation, integrating the intervention into clinical practice and clarifying expected time commitments and benefits at diagnosis is recommended. The low attrition rate (7.7%), with only one dropout in each group, indicated high feasibility and acceptance.^36^ All intervention group participants received needs assessment and cost-related health education, suggesting that these components could be integrated into clinical routines. However, over 30% of participants did not review the booklet after initial education. This was consistent with findings from Zhang et al., who observed similar issues in an educational intervention for cervical cancer screening,^37^ possibly due to the overwhelming amount of information patients received. Future interventions could incorporate follow-up reminders or digital resources to maintain engagement. Approximately 23.1%–46.2% of participants sought further details about financial assistance programs or personalized cost-related issues, finding the referral and personalized counseling helpful. This aligned with Kircher et al.’s findings, highlighting the necessity of tailoring financial intervention content to patients' individual needs.^38^

Regarding preliminary effects, both intervention and control groups experienced worsening FT at the 1-month follow-up, likely due to intensive treatments and associated costs after diagnosis. This aligned with previous reviews identifying increased treatment costs and reduced income as primary causes of FT.^5^^,^^19^ No significant between-group differences in FT were found post-intervention, consistent with Watabayashi et al.^31^ but not with Wheeler et al.,^39^ who reported significant improvements in COST-FACIT scores in a pre-post FN study. This might because our intervention did not provide direct, and practical financial assistance to participants. It is reassuring that the intervention significantly improved cost-related health literacy compared to the control group. Given the link between cost-related health literacy and FT,^40^ our intervention might improve FT through this modifiable variable. Future RCTs over longer durations are needed to validate this potential causal pathway. Though no participants in the intervention group delayed or forewent treatments due to costs, there were no significant post-intervention between-group differences in coping behaviors related to FT. Existing literature suggested behavior change was complex and might require longer time to capture shifts.^41^ The limited sample size and short duration of this pilot study might influence the evaluation of all domains of FT.^42^

Participants reported high satisfaction (median scores 23.5 out of 25) and perceived benefits from the intervention. They found personalized counseling via phone or WeChat acceptable and helpful, aligning with Kircher et al.’s findings on the feasibility of such delivery methods compared to in-person visits, which often face scheduling and transportation barriers.^38^ Future studies could explore other social media platforms for information delivery and ensure the intervention's feasibility and acceptability.^43^ Evidence suggested reading materials were valuable supplementary tools for information delivery.^10^^,^^44^ The booklet developed in this study enabled participants to easily review and identify appropriate coping strategies for FT when encountering problems after discharge. Based on participants' feedbacks, future booklets should include more information on medical insurance, financial assistance programs, and disease management.

### Implications for nursing practice and research

This study provides meaningful insights for future practice. The intervention's feasibility and acceptability indicate its potential for widespread integration into clinical settings, particularly in resource-constrained environments. Although the study did not observe significant changes in FT, its impact on health literacy shows potential to alleviate FT over time. By integrating FT assessment into cancer care, enhancing patients' cost-related health literacy, and providing access to financial assistance resources, cancer patients can be empowered to manage treatment-related expenses more effectively. It offers strategies at both institutional and individual levels to support financial well-being and improve patient outcomes.

### Limitations

This pilot study had several limitations. The small sample size and short follow-up period might limit the assessment of the intervention's effectiveness in mitigating all domains of FT. Data saturation may not have been fully achieved in semi-structured interview, which limits the depth of the findings. The single-institution setting in a metropolitan cancer center might not generalize findings to other regions, especially remote or underserved rural areas. Additionally, the lack of a cost-effectiveness analysis regarding the human resource investment for the navigator role may affect the assessment of the sustainability of implementing FN programs in routine clinical practice.

## Conclusions

Despite no significant between-group differences in FT, this pilot study suggests that an evidence-based FN program is feasible, acceptable, and beneficial in improving cost-related health literacy among patients with breast cancer in China. The insights gained from this study warrant further full-scale RCTs with larger sample sizes, longer follow-up periods and cost-effectiveness analysis to confirm these results and explore the intervention's effectiveness and sustainability in mitigating FT and improving other health outcomes.

## CRediT authorship contribution statement

**Xiaoyi Yuan**: Concept and design, Acquisition of data, Analysis and interpretation of data, Drafting and revision of the manuscript. **Liqin Chen**: Acquisition of data, Analysis and interpretation of data, Revision of the manuscript. **Yanling Sun**: Acquisition of data, Analysis and interpretation of data. **Yi Kuang**: Acquisition of data, Analysis and interpretation of data. **Junyi Ruan**: Acquisition of data. **Lichen Tang**: Critical revision of the paper for important intellectual content, Administrative, technical, or logistic support, Supervision. **Jiajia Qiu**: Critical revision of the paper for important intellectual content, Administrative, technical, or logistic support, Supervision. **Weijie Xing**: Concept and design, Critical revision of the paper for important intellectual content, Obtaining funding, Supervision. All authors have given their final approval of the manuscript. All authors had full access to all the data in the study, and the corresponding author had final responsibility for the decision to submit for publication. The corresponding author attests that all listed authors meet authorship criteria and that no others meeting the criteria have been omitted.

## Ethics statement

The study was approved by the Ethics Committee of Fudan University Shanghai Cancer Center (Approval No. 2312288-16) and was conducted in accordance with the 1964 Helsinki Declaration and its later amendments or comparable ethical standards. All participants provided written informed consent.

## Data availability statement

The data that support the findings of this study are available from the corresponding author, WX, upon reasonable request.

## Funding

This work was supported by the China Medical Board Open Competition Program (Grant No. 20-371, 2020); and the Youth Science Fund Project of National Natural Science Foundation of China (Grant No. 72004034, 2021). The funders had no role in considering the study design or in the collection, analysis, interpretation of data, writing of the report, or decision to submit the article for publication.

## Declaration of generative AI and AI-assisted technologies in the writing process

No AI tools/services were used during the preparation of this work.

## Declaration of competing interest

The authors declare no conflict of interest. Professor Weijie Xing, the corresponding author, serves on the editorial board of the *Asia–Pacific Journal of Oncology Nursing*. The article underwent standard review procedures of the journal, with peer review conducted independently of Professor Xing and their research groups.
